# How safe is BDSM? A literature review on fatal outcome in BDSM play

**DOI:** 10.1007/s00414-021-02674-0

**Published:** 2021-08-12

**Authors:** Anouk Schori, Christian Jackowski, Corinna A. Schön

**Affiliations:** Institute of Forensic Medicine, University of Bern, Bühlstrasse 20, 3012 Bern, Switzerland

**Keywords:** Erotic asphyxiation, Strangulation, Sexual activity, Non-natural death, Sadism, Masochism

## Abstract

A noteworthy number of people are interested in BDSM (bondage and discipline, dominance and submission, sadism, and masochism). Fatal outcomes while participating in BDSM activities occur. The aim of this literature review is to give a better insight into potential dangerous BDSM play by summarizing published data on BDSM fatalities. A literature search was conducted. It was searched for non-natural death related to BDSM activity. Seventeen cases were found. The age of the deceased ranged between 23 and 49 years (mean age 34.9 years). Strangulation in the course of erotic asphyxiation was the most common cause of death (88.2%). In 13 cases, a toxicology report for the deceased was mentioned, of which in eight cases (61.5%) toxicology analysis was positive. In four of these cases, the BDSM partner was also tested positive with the same substance. Drugs or alcohol was involved in 64.3% of fatal BDSM play. In nine cases, the level of experience in BDMS activity of the deceased and the partner was described, and in all of them, the deceased and the partner were not new to BDSM play. Fatal outcomes of BDSM plays are rarer than autoerotic fatalities and natural deaths related to sexual activities. Safeguards and education on medical aspects exist in the BDSM communities. If they are followed by the practitioners, the risks of BDMS play can be reduced. Cases of non-natural death connected to BDSM are rare incidents and can be prevented.

## Introduction

BDSM is a combination of the abbreviations of bondage and discipline (BD) and dominance and submission (DS) along with sadism and masochism (SM). BDSM interactions are sexual practices between two or more individuals. They include sexual activities, which create an intense sensation by physical restriction, and/or aspects of power exchange. In BDSM, one party is taking on the role of the dominant who temporally has power over the submissive [1]. The practitioners agree on their roles and that physical injury may be applied to increase pleasure. BDSM is based on the concept of consent between the individuals involved [2].

Studies show that there is a noteworthy number of people interested in BDSM. An internet-based study with 2021 participants conducted in the USA in 2015 focused on a broad range of sexual interests [3]. It showed that 20% of the participants had experiences with tying/being tied up, about 30% in spanking, and 13% in playful whipping. The results of a systematic scoping review of epidemiological data on BDSM point out that BDSM-related fantasies are common (40–70%) [4]. FetLife, a free social network for people interested in BDSM, reported around 20,000 members living in Switzerland [5].

A recently published case collection described three cases of fatal incidents related to consensual BDSM activity [6]. These non-natural deaths associated with BDSM activity raised several questions concerning the topic: How frequent is a fatal outcome in BDSM play? What are the risks of BDSM play? How well are participants informed about safety issues and medical aspects in those potentially dangerous activities? Is fatal BDSM play a consequence of a lack of knowledge or education of private and professional practitioners?

This paper aims to provide data of published case reports involving the fatal outcome of BDSM play. The focus lies on the causes of death, the education of BDSM practitioners, and the safety issues in BDSM play.

## Materials and methods

A search for case reports and studies was performed by using the databases PubMed, Embase (OvidSP), and Medline (OvidSP) for German and English articles. The search focused on non-natural death involving BDSM activity. The following search terms were used: BDSM and death OR sadism and death OR masochism and death OR erotic death OR asphyxiophilia OR sexual asphyxia OR kink and death. Excluded were all papers solely related to autoerotic death, as the interest was laid on fatal outcome during consensually agreed BDSM activities involving more than one person.

Reviewing the case reports revealed that comparable cases of asphyxia deaths were described differently. As the causes of death in BDSM fatalities are the essential aspect of this review, we find it important to clarify the definitions of certain terminologies. Different variations in the classifications of asphyxia and definitions of its subtypes can be found in the literature. We used the classification by Sauvageau and Boghossian [7] to categorize the causes of death in the compared cases. They classify asphyxia into four categories: strangulation, suffocation, mechanical asphyxia, and drowning. The definitions of terms, based on Sauvageau and Boghossian [7] and Bauer et al. [6], are clarified in Table 1.

**Table 1:** Definitions of the different forms of asphyxia

Term	Definition
Strangulation	Superordinate term for manual strangulation, ligature strangulation, and hanging
Manual strangulation	Form of strangulation: compression on the neck by hands, forearms, or other limbs
Ligature strangulation	Form of strangulation: pressure on the neck applied by a ligature tightened by a force other than the body weight
Hanging	Form of strangulation: compression on the neck applied by a ligature constricted by the gravitational weight of the body or part of the body
Suffocation	Broad term for different types of asphyxia such as smothering, choking, and confined spaces/entrapment/vitiated atmosphere associated with deprivation of oxygen
Smothering	Blockage of the air passages above the epiglottis, including the nose, mouth, and pharynx
Choking	Blockage of the air passages below the epiglottis
Confined spaces/entrapment/vitiated atmosphere	Asphyxia caused by an exposition to an inadequate atmosphere with reduction of oxygen or displacement of oxygen by other gases
Mechanical asphyxia	Superordinate term for traumatic asphyxia and positional asphyxia
Traumatic asphyxia	Asphyxia caused by the position of an individual which leads to the inability to breath
Positional asphyxia	Asphyxia caused by external chest compression by a heavy object
Drowning	Asphyxia by immersion in a liquid

## Results

Between 1986 and 2020, 14 case reports and one study on non-natural death associated with sexual activities including BDSM play were published. A 25-year medicolegal postmortem study from 1993 to 2017 on 16,437 autopsies was conducted in Germany. Of the 74 cases of non-natural death associated with sexual activity, 3 cases (4.05%) were associated with BDSM activities [8]. The 17 cases are described in Table 2.

**Table 2:** Reported cases associated with BDSM activities; *F*, female; *M*, male

Case *#*	Age	Sex	Cause of death	BDSM play	Experience in BDSM	Deceased’s relationship with partner	Toxicology	Reference *#*
1	32	M	Manual strangulation	Strangulation with the forearm of sexual partner	Not noted	Sexual partner (M)	Negative (deceased), partner not noted	Michalodimitrakis et al. [37]
2	25	F	Ligature strangulation	Rope around the neck for a sexual partner to control, deceased’s arms and legs fixed, towel in the mouth with a ligature consisting of a scarf and belt	Not noted	Sexual partner (M)	Not noted	Baik and Uku [38]
3	30	F	Manual strangulation	Strangulation with hands and manually closed respiratory orifices by the partner	Yes (both)	Sexual and BDSM partner (M)	Alcohol (both)	Oklota et al. [10]
4	41	M	Ligature strangulation	Rope around the neck for a sexual partner to control, deceased’s arms and legs were tied	Deceased yes, partner not noted	Sexual partner (M)	Negative	Oklota et al. [10]
5	49	M	Suffocation, oronasal occlusion	Mouth sealed with tape, arms and legs fixed, partner pressing hands on the mouth and nose	Deceased was a member of a BDMS community and has been known for participating in breath control plays, partner not new to breath control play	BDSM partner (F)	Negative	Madea and Hagemeier [11]
6	23	F	Incomplete hanging	Shibari bondage practice, double hanging with rope at least twice around the neck of the deceased	Deceased and female partner had attended classes in BDSM, third partner (observer) experience in BDSM not noted	BDSM partner (F), observer (M), all three met online	Moderate quantity of alcohol and cannabinoids (both)	Roma et al. [12]
7	27	F	Manual strangulation	Strangulation with hands by sexual partner	Not noted	Sexual partner (M)	Drugs (not clarified which substances), (both)	Sendler [9]
8	31	F	Ligature strangulation	Throttling with belt	Not noted	Boyfriend	Alcohol (partner), deceased not noted	Sendler [9]
9	26	F	Manual strangulation	Strangulation with hands, with a bag placed overhead	Deceased not noted, client not new to breath control play	Client (M) of sex worker	Rape pill substances (not clarified which substances) (deceased), partner not noted	Sendler [9]
10	30	F	Manual strangulation	Strangulation with a hand and closing respiratory orifices with a cloth	Deceased participating in breath control plays for some years, partner has been involved in BDSM plays before, but new to breath control play	Sexual and BDSM partner (M)	Negative (both)	Sendler [9]
11	41	M	Ligature strangulation	Rope around the neck for a sexual partner to control, deceased’s arms and legs fixed	Deceased interested in breath control plays for two years, partner not noted	Sexual partner (M)	Negative (both)	Sendler [9]
12	Not noted	M	Ligature strangulation	Strangulation with a throttling device	Yes (both)	Not noted	Not noted	Bunzel et al. [8]
13	Not noted	M	hanging	Strangulation with hanging	Yes (both)	Not noted	Not noted	Bunzel et al. [8]
14	Not noted	F	Severe hemorrhage combined with alcohol intoxication	Vaginal insertion of foreign objects, one was an inflatable balloon pump	Yes (both)	Not noted	Alcohol (both)	Bunzel et al. [8]
15	46	M	Ligature strangulation	Straightjacket and headgear with gag, tied to a wooden board with shoelaces fastened around neck or headgear	Yes (both)	Dominatrix (F)	Alcohol (deceased), cocaine (partner)	Bauer et al. [6]
16	49	M	Strangulation, compatible with hanging	Incomplete hanging with collar and chain attached to the ceiling, whip play by partner	Deceased yes, partner not known	Sexual partner (M)	Cannabinoids (deceased), partner not noted	Bauer et al. [6]
17	38	M	Strangulation compatible with hanging	Chain around the neck, tied to a column, dildo in mouth, electrical stimulation of genitals and nipples	Yes (both)	Dominatrix (F)	Acetone, 2-propanol (components of “poppers”) (deceased), partner negative	Bauer et al. [6]

### Epidemiological data

Eight female and nine male deceased were documented. In 14 of the 17 cases, the age of the deceased was given; the age ranged was between 23 and 49 years (mean age 34.9 years). In five cases, the participants in the BDSM play were of the same sex. In one of these cases, a third person of the opposite sex as the two participants attended as an observer.

### Experience in BDSM

In nine cases, the deceased and the partner were not new to BDSM activities. In two cases, the BDSM practitioners instructed their partners about breath control play and taught cardiopulmonary resuscitation before carrying out BDSM activities [9, 10]. In two of these cases, the partners were dominatrices and the decedents were not new to BDSM activities [6]. In one of the nine cases, the deceased instructed his partner with a manual and pictures on the breath play; however, no safeword was agreed upon. The deceased had been known for his preferences for breath control play and had offered his female partners money if they succeed to make him unconscious [11]. In the other eight cases, either only one participant was experienced in BDSM activities or the level of experience of one or both participants was not noted.

### Relationship between BDSM partners

The relationship status between the decedents and their partners was various. In four cases, the deceased and their partner have been meeting up for BDSM activities for some time. In two cases, the deceased were clients of dominatrices; in both cases, it was not the first meeting between the client and the dominatrix [6]. In one case, the deceased was a prostitute. It was not noted if the partner was a frequent client or if they met for the first time [9]. In another case, the two participants and the observer met online. The incident occurred at their first meeting [12].

### Cause of death

The most common cause of death in the course of erotic asphyxiation is strangulation (*n* = 15). The causes of death in fatal BDSM play are shown in Table 3. The double hanging with traditional Japanese rope bondage technique (shibari), performed by two women, led to the death of one participant [12]. They were tied to each other in a sort of pendulum as the ropes were slung over metal tubes in the basement and passed around the participants’ necks. When one participant went down, the other went up. As one woman fainted, the partner remained suspended. The woman, who fainted in the play, died of mechanical asphyxia while her partner suffered respiratory failure and was in a coma. A male observer was present but did not manage to stop the state of equilibrium. He had no knife on him.

**Table 3:** Causes of death in fatal BDSM play

Cause of death	*n*
Strangulation	15
Manual	5
Strangulation with hands, respiratory orifices closed with cloth/hands	2
Strangulation with hands	1
Strangulation with hands and bag over head	1
Strangulation with forearm	1
Ligature	6
Controlling rope during sexual intercourse	3
Throttling	2
Tied to a wooden board with shoelaces around neck/headgear	1
Hanging	4
Incomplete hanging	3
Incomplete hanging, double hanging	1
Suffocation/oronasal occlusion	1
Hemorrhage combined with alcohol intoxication	1

### Toxicological examinations

Toxicology reports of the decedents showed presence of alcohol (*n* = 4), cannabinoids (*n* = 2), acetone, 2-propanol (components of “poppers”) (*n* = 1), “rape pill” (*n* = 1), and “drugs” (*n* = 1) (no further explanation for “rape pill” and “drugs” was given). Negative reports for the deceased were present in five cases. In four cases, the partner involved in the BDSM play was positively tested with the same substances as the deceased. In one case, the partner was positively tested with a different substance. In another case, the partner was under the influence of alcohol and the toxicology analysis of the deceased was not mentioned. In three cases, the toxicology analysis of the deceased and the partner was not mentioned or had not been made.

## Discussion

### BDSM activities

BDSM actions embrace a broad range of practices. There are more common activities, for example, rope bondage and flogging (use of multi-tailed whip) to rarer fetishes [13]. The wide variety of BDSM activities, as shown in Table 4, can be divided into impact play, sensation play, rough body play, genitals, sex and bodily fluids, sensory deprivation, bondage, scene or relationship dynamics, role play and psychological play, and body modification [14]. Although penetrative sex acts can be included in BDSM play, sexual intercourse does not necessarily occur [15]. Physical health complications such as bruising and musculoskeletal injuries due to whipping, spanking, and flogging; broken skin leading to infections or nerve damage due to bondage or handcuffs; burns due to hot wax play; blood-borne pathogen exposure due to needling or temporary piercings; fainting due to vasovagal response to pain; and emotional intensity may occur in BDSM plays [16]. Wiseman [1], a prominent BDSM community author, explains in his BDSM beginner’s book *SM 101: A Realistic Introduction* the risk of breath control play and clarifies that practitioners can be taught how to participate in such activities safely. However, the health risks of breath play can never be eliminated. Edge plays are BDSM activities with a high physical or psychological risk. Sexual asphyxia or breath control play can be named as one of them. Breath control play is very controversial in the BDSM scene, and some BDSM communities ban members from engaging in breath control play [17]. In general, BDSM practitioners seem to be well informed on potential injuries. Still, 44% of interviewed practitioners have consulted a medical professional for BDSM-related matters. Because of fear of discrimination, BDMS practitioners tend not to seek medical advice, when it is needed [16].

**Table 4:** Diversity of BDSM activities—explanations (Taormino [14])

Impact play	Spanking, caning, slapping, flogging, florentine flogging (two-handed style of flogging), whipping
Sensation play	Clips, clamps, pinching, hot wax, knife play, electricity play, tickle torture, cupping, fire cupping, fire play
Rough body play	Slapping face, hair pulling, spitting, punching, wrestling, biting, scratching
Genitals, sex, and bodily fluids	Vaginal fisting, anal fisting, rough sex, cock and ball play, genital play, genitorture, sounds, enema, watersports/golden showers (urine)
Sensory deprivation	Sight deprivation, sound deprivation, scent deprivation, gagging, mummification, breath control play
Bondage	Rope bondage, cuffs, metal bondage, Japanese bondage, suspension, chastity device, predicament bondage, mummification, confinement
Scene or relationship dynamics	Master/slave, domestic servitude, sexual service, personal service, 24/7 D/s (permanent dom/sub-role)
Role play and psychological play	Animal role play, age play, taboo play, psychological manipulation, interrogation, objectification, humiliation, kidnapping, orgasm control/denial, forced cross-dressing, medical play
Body modification	Play piercing/temporary piercing, suture play, stapling, saline inflation, permanent piercing, cutting, branding

The fatal outcome in BDSM play seems to be very rare. There are 14 published case reports and one organized study on non-natural death associated with sexual activity. The study by Bunzel et al. showed that of 16,437 autopsies from 1993 to 2017, 0.018% (3 cases) were non-natural deaths related to sexual activity involving BDSM. On the other hand, 0.13% of cases were non-natural deaths in autoerotic practices [8]. Similar numbers of autoerotic fatalities can be found in other studies conducted in Germany (0.1%) [18] or Canada (0.2%) [19]. The incidences of autoerotic deaths per million inhabitants per year have been reported to be 0.3 in Australia and 0.14 in Sweden [20], 0.5 in Germany [18], and 0.2–0.5 in Canada [21]. The results of Bunzel et al. on BDSM fatalities can provide a rough point of reference for the incidence of fatal outcome in BDSM play [8].

As for natural death related to sexual activities, a 45-year mortality study conducted in Germany showed a rate of 0.26%. The men’s mean age was 57.2 years, and the women’s mean age was 45 years. The most common cause of death was coronary heart disease, followed by myocardial infarction and reinfarction [22]. A few cases are described on natural death in association with autoerotic activities [23–26], but no cases on natural death connected to BDSM play. A reason might be the age of BDSM practitioners. People under the age of 50 years tend to be more interested in BDSM activities than the older population [4, 27]. This seems to correspond with the mean age of the here presented cases, which was 34.9 years.

### Causes of death

The most prominent cause of death in BDSM fatalities emerging from the 17 reported cases was strangulation (88.2%). It can be differentiated between ligature and manual strangulation and hanging; ligature strangulation was documented as the most frequent cause of death. In one case, the cause of death was severe hemorrhage caused by the vaginal insertion of foreign objects [8]. Strangulation followed by intoxication is also the most frequent cause of death in the course of autoerotic practices [8, 21]. Traumatic injury, perforation, and electrocution are named as other causes of death in autoerotic fatalities, but they are rarer than strangulation [20].

Care is necessary when describing different causes of asphyxia and in particular strangulation. In two cases [9, 10], the deceased was strangulated with hands by the partner. The authors described the causes of death strangulation as sexual choking. The usage of the term choking in this context is incorrect, as choking by definition is asphyxia by obstruction of the air passage and not external pressure applied on the neck [7]. Sexual choking with hands should better be described as strangulation with hands and therefore as a form of manual strangulation. In the compared cases, the classification of asphyxia and also the definitions of subtypes were not consistent. The model of classification and definitions by Sauvageau and Boghossian [7] was used to compare the different cases and leads to the results we presented in this review. The definitions of the subtypes of asphyxia are described in Table 1. It is important when describing BDSM fatalities to use forensic terminology and definitions and not general BDSM terms.

A difficulty concerning BDSM fatalities is the forensic case work with the aim to reconstruct the fatal event. Especially in these delicate situations, to prevent misinterpretations, the forensic experts must have in mind that the original situation of the fatality could have been changed. Except for medical interventions like resuscitation measures by the partner and/or medical professionals, it is possible that the partner could have changed certain features of the scene, e.g., because of feeling ashamed or being afraid of the possible consequences. Sex toys and other BDSM equipment could have been removed or the other person could have even mimicked an autoerotic death and fled the scene. Mostly, there are no other witnesses than the probably shocked sexual partner who is not able or does not want to give a detailed explanation of the situation. Statements of the present participant(s) can be inexact or contradictory due to emotional stress after witnessing the other person’s death [6]. Also, incorrect shame-associated statements from relatives about the deceased’s sexual behavior or interest in BDSM can lead to wrong conclusions [8]. In two of the described cases, there were various video materials found on the deceased’s computers [9, 10]. The films contained breath control play of hetero- and homosexual partners as well as autoerotic practices. Such videos material on BDSM activities can offer assistance to reconstruct fatal events. Therefore, forensic experts should search for electronic or printed material on BDSM activities as well as hidden cameras at the scene.

### Toxicology

The use of alcohol and drugs can play a relevant role in sexual activity. In 58% of autoerotic fatalities, the toxicology reports were negative or a therapeutic presence of drugs was found [21]. Bunzel et al. report only 36% negative toxicology reports for autoerotic fatalities and could not confirm the use of nitrites (poppers) in any case [8]. An online, cross-sectional global drug survey with 22,289 responses reported alcohol as the most common drug to have sex on, followed by cannabis and MDMA. For homosexual men, the fourth most frequently drug used was poppers, while in the other groups (heterosexual men, homosexual and heterosexual women) it was rarely reported [28]. BDSM communities disclaim the use of drugs and alcohol during plays. Drugs and alcohol can affect the practitioner’s ability to participate in a BDSM play with the needed safety precautions [17]. Wiseman states that performing BDSM activities while being drunk or on drugs is unsafe and irresponsible [1]. The use of mind-altering substances during BDSM play is controversially discussed within the BDSM communities, and many BDSM organizations have specific rules against the use of substances [17]. In only 13 of the 17 described cases, a toxicology report for the deceased was mentioned. In 8 of the 13 cases (61.5%), the toxicology analysis was positive. These results show that fatalities correlate with the usage of alcohol and drugs. The numbers correspond with the results from Bunzel et al. for autoerotic fatalities [8].

### BDSM communities and safeguards

In 9 cases, the deceased and the partner were not novices to BDSM activities. In two of these cases, the partner was a dominatrix. In three cases, instructions were given, and cardiopulmonary resuscitation was discussed before starting BDSM play. In all three cases, cardiopulmonary resuscitation was performed by the partner. Our results show that despite being informed about BDSM activities and the presence of medical knowledge, BDSM play can still have a fatal outcome. In the USA, about 85% of the BDSM practitioners said that they received some of their BDSM education from a mentor of the scene, whereas only about 9% stated that they did not gain their knowledge from a mentor directly. Of 1405 asked BDSM practitioners in the USA, 79% belonged to a BDSM organization [29, 30]. BDSM communities developed clear ethical values and guidelines to minimize risk and create an effective way of mentoring and educating novices to BDSM. They offer classes and workshops on dungeon (public or private place for BDSM gatherings) safety, how to classes on techniques and skills, setting the scene, or negotiation and aftercare, to mention just a few [13]. Meetings for novice practitioners contain basic first aid information and more specialized information such as changes in breathing patterns and how to handle emergencies [31].

BDSM practitioners use a safeword, a prearranged word, or a gesture that can be signaled by one of the practitioners with the wish to end the play. It is a way to communicate the physical and emotional discomfort [2]. This is part of the overall principle in the BDSM scene, which is referred to as safe, sane, and consensual (SSC). The term risk-aware consensual kink (RACK) is preferred by certain BDSM practitioners, as it does not use the word “sane,” which can implicate negative stereotypes [15]. Also, there exists a universal safeword system called the “traffic light” system with the opportunity to point out whether the intensity is great (green), slowing down (yellow), or stopping the play (red). A standardized safeword system has the benefit that it can be used as a “house safeword” in most of the BDSM communities [32]. BDSM communities attach great importance to education and mentoring, and therefore, educational components are a significant part of the organization’s activities [29, 33]. It was not possible to show a correlation between the agreement on a safeword and fatal outcome with our results. In only one case, it was mentioned that no safeword was used; in all other cases, it was not noted.

In two cases, the deceased was a client of a dominatrix [6]. Professional dominatrices provide their services in private dungeons. Male professional dominants are rare [34]. Only a few professional dominatrices offer intercourse, oral sex, or manual gratification to their clients [35]. Professional dominatrices gain their knowledge and BDSM training, mainly by “hands-on” mentoring [34]. They have to learn to minimize psychological and physical risks and how to react if psychological reactions or physical and medical issues occur [34, 36]. Professional dominatrices are well aware of the risks of BDSM plays and acquire knowledge about their client’s physical and psychological responses [34]. Organizations within the BDSM scene offer classes on these topics specifically for professional dominatrices [36].

It can be agreed with Lee et al. that “(…) it is inaccurate to characterize all BDSM activities as extremely dangerous games.” Safeguards exist, and fatalities can be prevented if they are followed [17]. Our results show that strangulation in the course of erotic asphyxiation is the most common cause of death in fatal BDSM play. BDSM practitioners should be aware of the potential risks in erotic asphyxiation practices. We emphasize to have an emergency or backup plan when practicing BDSM, for example, to have specific tools close at hand to free participants from bondage constructions or the option to quickly bring in another person when needed. We share the opinion about the importance to include accurate information about the risks of BDSM activities in sexual education as an attempt to prevent these fatalities. People should understand the potential risk of unsafe BSDM play, including the danger of combining alcohol or drugs and BDSM activities and that it is essential to learn how to avoid fatal incidents when participating in such activities [8, 12].

## Strengths and limitations

One limiting factor of this study is the low number of published research and case studies on BDSM fatalities. Whereas for autoerotic deaths, an abundance of case reports and studies has been published; only 17 cases related to fatal outcomes in BDSM play were found. A second noteworthy limitation is restricted accessibility to BDSM practitioners by fear of stigmatization and, additionally, for professionals, a fear to reveal a lack of medical knowledge and sanitary precautions. BDSM is still a taboo subject. Despite this limitation, the paper presents a collection of BDSM-related deaths and outlines the circumstances. Furthermore, this research gives an overview of how BDSM practitioners are educated and the possible safeguards in BDSM play.

## Conclusion

BDSM practices and related fantasies are not uncommon among the general population. Strangulation in the course of erotic asphyxiation is the leading cause of death in BDSM play. The usage of drugs and alcohol or the lack of knowledge in BDSM activities correlates with such fatalities. BDSM fatalities are rare compared to autoerotic deaths or natural deaths related to sexual activities. It is essential to continue further studies on BDSM-related deaths and injuries to help overcome difficulties of forensic assessment and reconstruction of cases of non-natural death connected to BDSM.

## Data Availability

Not applicable.
